# Postnatal Changes of Somatostatin Expression in Hippocampi of C57BL/6 Mice; Modulation of Neuroblast Differentiation in the Hippocampus

**DOI:** 10.3390/vetsci10020081

**Published:** 2023-01-21

**Authors:** Dae Young Yoo, Woosuk Kim, Hyo Young Jung, In Koo Hwang

**Affiliations:** 1 Department of Anatomy and Convergence Medical Science, Institute of Health Science, Tyrosine Peptide Multiuse Research Group, College of Medicine, Gyeongsang National University, Jinju 52727, Republic of Korea; 2 Department of Anatomy, College of Veterinary Medicine, Konkuk University, Seoul 05029, Republic of Korea; 3 Department of Veterinary Medicine & Institute of Veterinary Science, Chungnam National University, Daejeon 34134, Republic of Korea; 4 Department of Anatomy and Cell Biology, College of Veterinary Medicine, and Research Institute for Veterinary Science, Seoul National University, Seoul 08826, Republic of Korea

**Keywords:** somatostatin, postnatal, hippocampus, neuroblast differentiation

## Abstract

**Simple Summary:**

Somatostatin expression in the hippocampus is transiently increased during the postnatal development of the hippocampus and decreased after P21. This study suggests that expression of SST is closely associated with postnatal neuroblast differentiation in the hippocampus.

**Abstract:**

(1) Background: Somatostatin (SST) exhibits expressional changes in the brain during development, but its role is not still clear in brain development. (2) Methods: We investigated postnatal SST expression and its effects on hippocampal neurogenesis via administering SST subcutaneously to P7 mice for 7 days. (3) Results: In the hippocampal CA1 region, SST immunoreactivity reaches peak at P14. However, SST immunoreactivity significantly decreased at P21. In the CA2/3 region, the SST expression pattern was similar to the CA1, and SST-immunoreactive cells were most abundant at P14. In the dentate gyrus, SST-immunoreactive cells were most abundant at P7 and P14 in the polymorphic layer; as in CA1-3 regions, the immunoreactivity decreased at P21. To elucidate the role of SST in postnatal development, we administered SST subcutaneously to P7 mice for 7 days. In the subgranular zone of the hippocampal dentate gyrus, a significant increase was observed in immunoreactivity of doublecortin (DCX)-positive neuroblast after administration of SST.; (4) Conclusions: SST expression in the hippocampal sub-regions is transiently increased during the postnatal formation of the hippocampus and decreases after P21. In addition, SST is involved in neuroblast differentiation in the dentate gyrus of the hippocampus.

## 1. Introduction

The hippocampus, important for learning and memory, is part of the medial temporal lobe [1]. Experiments involving the selective removal of the hippocampus have shown that the hippocampus is closely involved in contextual and spatial memory processes [2]. The hippocampus has been focused on by researchers because neurogenesis occurs in the subgranular zone of the hippocampal dentate gyrus throughout life [3,4]. In the normal development of the rat brain, hippocampal structures are formed in a particular sequence by neurogenetic gradients; pyramidal cells in the hippocampal Cornu Ammonis (CA) originate after large cells in the strata lacunosum–moleculare, oriens, and radiatum, whereas granule cells in the dentate gyrus generate after large cells in the molecular and polymorphic layers [5]. In the CA of the hippocampus, pyramidal cells are generated earlier than granule cells in the dentate gyrus [6]. Fifteen percent of the granule cells in the rat dentate gyrus are formed prenatally, and massive granule cells proliferate during the first postnatal week [7]. In mice, the formation of granule cells continues until the third postnatal week [8,9].

Somatostatin (SST), a growth hormone inhibiting factor, is expressed in various peripheral organs and the central nervous system (CNS) [10,11]. Neuroendocrine, inflammatory, and immune cells produce SST in response to multiple factors, such as nutrients, neurotransmitters, hormones, cytokines, and growth factors [11]. During brain development, SST shows expressional changes in several sub-regions [12,13], and it has trophic factor-like activity and is involved in modulating neuronal regeneration and differentiation [12,14]. In the rodent hippocampus, SST-expressing interneurons are detected in the dentate gyrus, CA1, and CA3 regions [15], and SST acts as a neurotransmitter involved in tuning neuronal activities and modulating synaptic plasticity [16,17]. The number of SST-positive neurons in the hippocampal formation decreases with age, along with a decrease in cholinergic varicosities [18].

It has been reported that intensive hippocampal neurogenesis occurs during the early postnatal weeks [19], and SST is expressed in several regions of the developing brain [12,13]. However, few studies focused on expressional changes of SST during postnatal development of the hippocampus and the correlation between SST expression and hippocampal neurogenesis. Therefore, we investigated the ontogenetic SST expression in sub-regions of the mouse hippocampus at various early postnatal stages. We also investigated the effects of subcutaneous injections of SST on neurogenesis in the hippocampal dentate gyrus.

## 2. Materials and Methods

### 2.1. Experimental Animals

C57BL/6 male mice were used in this study, and the animals were sacrificed at 4 time points [postnatal day 1 (P1), 7 (P7), 14 (P14), and 21 (P21), *n* = 30]. The day of birth was considered P0. A maximum of two animals from one liter were used for each age group, and animals of same age were from at least two different litters. All procedures with animals were followed by the guidelines (NIH Guide for the Care and Use of Laboratory Animals, NIH Publication No. 85-23, 1985, revised 1996) and were approved by the Institutional Animal Care and Use Committee at Seoul National University (approval no.: SNU-140313-1) and Kangwon National University (approval no.: KW160407-2). In the present study, all experiments were performed in a manner that minimized animal numbers and suffering caused by the procedures.

### 2.2. Tissue Processing

Experiment I: The animals in each group (P1, P7, P14, and P21; *n* = 5 in each group) were anesthetized in an induction chamber with 5% isoflurane (Baxter, Deerfield, IL, USA) because of higher MAC in 10-day-old pup than in adult mice [20,21]. The brains were removed and fixed in 4% paraformaldehyde at 4 °C for 24 h. After dehydration in graded concentrations of alcohol, the brains were embedded in paraffin. They were sectioned coronally with a 3 µm thick microtome (Leica Microsystems GmbH, Wetzlar, Germany).

Experiment II: The animals used in this experiment consisted of 2 groups (*n* = 5 in each group): vehicle (0.9% saline)-treated and SST (Sigma-Aldrich, St. Louis, MO, USA)-treated groups. At P7, vehicle and SST (0.25 mg/kg body weight) were subcutaneously administered once a day for 7 days. This was because P7 is a dynamic period in the usage of P7 rat and mouse pups to investigate perinatal scenarios [9,22,23]. Extravascular leakage into the brain parenchyma is observed until P12, due to low expression of pericytic desmin, important for astrocyte end-feet distribution, until P12 [24]. Thereafter, the brain was processed as described above.

### 2.3. Cresyl Violet Staining

The procedures for cresyl violet staining were carefully conducted under the same conditions to demonstrate the morphological changes of structures in the hippocampus as described in a previous study [9,25]. The brain sections were rehydrated in graded concentrations of alcohol and incubated in 0.1% cresyl violet solution for 15 min, and removed the excessive cresyl violet dye in 70% ethanol. Thereafter, the sections were dehydrated and mounted with Canada Balsam.

### 2.4. Immunohistochemistry for SST, Ki67, and Doublecortin

The procedures for immunohistochemistry were carefully performed under the same conditions to evaluate differences between the groups as described in previous studies [9,25]. The brain sections were rehydrated in graded concentrations of alcohol and quenched endogenous peroxidase activity with 0.3% hydrogen peroxide (H_2_O_2_) in phos-phate-buffered saline (PBS). For retrieving antigens in tissues, the brain sections were heated in citrate buffer (pH 6.0) with the retriever (2100 Antigen Retriever, Prestige medical, Lancashire, UK). Additionally, the slides were then cooled at 25 °C and washed in PBS. For protein blocking, the sections were incubated in 5% normal goat serum for 30 min. For primary antibody binding, the tissues were incubated in diluted rat anti-SST (1:100, EMD Millipore, Temecula, CA, USA), rabbit anti-Ki67 (1∶500; Abcam, Cambridge, UK, a marker for proliferating cells), or rabbit anti-doublecortin (DCX, Abcam, a marker for developing neuroblasts) antibodies for 48 h at 4 °C. Thereafter, they were exposed to biotinylated goat anti-rat IgG or goat anti-rabbit IgG (1:200, Vector Laboratories, Burlingame, CA, USA), streptavidin peroxidase complex (Vector Laboratories, Burlingame, CA, USA), and then visualized with 3,3′-diaminobenzidine tetrahydrochloride (Sigma-Aldrich) in 0.1 M Tris-HCl buffer (pH 7.4).

### 2.5. Double Immunofluorescence

Double immunofluorescence staining was performed to confirm the colocalization of SST and glial fibrillary acidic protein (GFAP) or SST and neuronal nuclei (NeuN) in the hippocampal sub-regions as described in the previous study [25]. Sections were incubated in the mixture of rat anti-SST (1:50, EMD Millipore)/rabbit anti-GFAP (1:500, Abcam) or mouse anti-NeuN (1:100, EMD Millipore) for 48 h at 4 °C. After washing three times with PBS, the tissues were incubated in a mixture of FITC-conjugated donkey anti-rat IgG (1:600, Jackson ImmunoResearch, West Grove, PA, USA) and Texas red-conjugated donkey anti-mouse IgG (1:600, Jackson ImmunoResearch) or Texas red-conjugated donkey anti-rabbit IgG (1:600, Jackson ImmunoResearch) for 2 h at room temperature. The immunofluorescence was detected by the confocal microscope (LSM510 META NLO, Carl Zeiss, Göttingen, Germany).

### 2.6. Data Analysis

In all the groups, SST and DCX immunoreactivities were measured by an image analysis system. SST and DCX immunoreactivities were expressed as the relative optical density (ROD) using the formula: ROD = log(256/mean gray level). The background value in unlabeled portions was subtracted for correction using ImageJ 1.53 (National Institute of Health, Bethesda, MD, USA). The ROD for SST was expressed as a percentage compared to the P7 group, and for DCX was expressed as a percentage compared with the vehicle-treated group. ROD and cell counts were averaged using 5 sections from each animal.

The data for SST immunoreactivities are expressed as the means of values obtained for each experimental investigation. One-way analysis of variance followed by the Tukey’s multiple comparisons test were performed to analyze statistical differences among the groups using the GraphPad Prism 9.41 (GraphPad Software, Inc., La Jolla, CA, USA). Statistical significance was considered at *p* < 0.05.

In the hippocampal dentate gyrus, Ki67-positive cells were measured using Optimas 6.5 (CyberMetrics, Scottsdale, AZ, USA). The Ki67-stained images were converted to a gray image, and Ki67-positive cells were automatically counted by the intensity of the Ki67 immunostaining. The data are expressed as the means of the values, and Student’s *t*-test was performed to elucidate the effects of SST on Ki67 and DCX expression in the mouse hippocampal dentate gyrus. Statistical significance was considered at *p* < 0.05.

## 3. Results

### 3.1. Double Immunofluorescence

**Figure 1:**
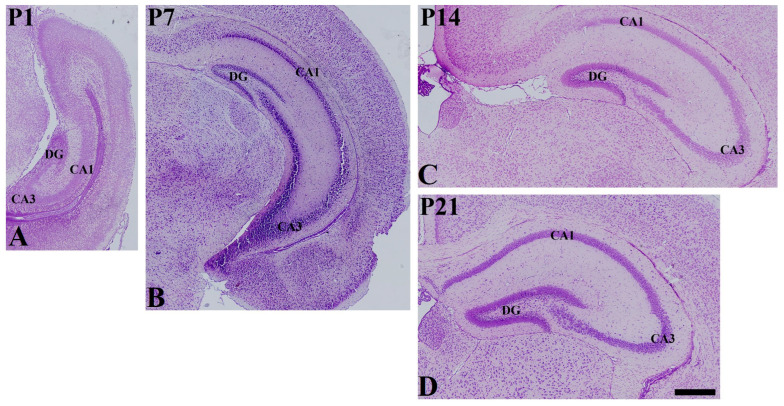
Cresyl violet staining in the mouse hippocampus at P1 (**A**), P7 (**B**), P14 (**C**), and P21 (**D**). DG dentate gyrus. Scale bar = 400 μm.

In the P1 group, cresyl-violet-stained cells showed thick lamina in the stratum pyramidale of CA1 and CA3 region. In addition, the three layers of lamination were not clear in the dentate gyrus (Figure 1A). In the P7 group, less abundant pyramidal cells were found in the stratum pyramidale of CA1 and CA3 region. In the dentate gyrus, the granule cell layers were distinguished from other layers (Figure 1B). In the P14 and P21 groups, clear lamination of hippocampal CA1 and CA3 region and dentate gyrus were found, and fewer cresyl-violet-stained cells were found in stratum radiatum and oriens of CA1 and CA3 region as well as the polymorphic layer of the dentate gyrus (Figure 1C,D).

### 3.2. Postnatal Expression of SST in the Hippocampus

**Figure 2:**
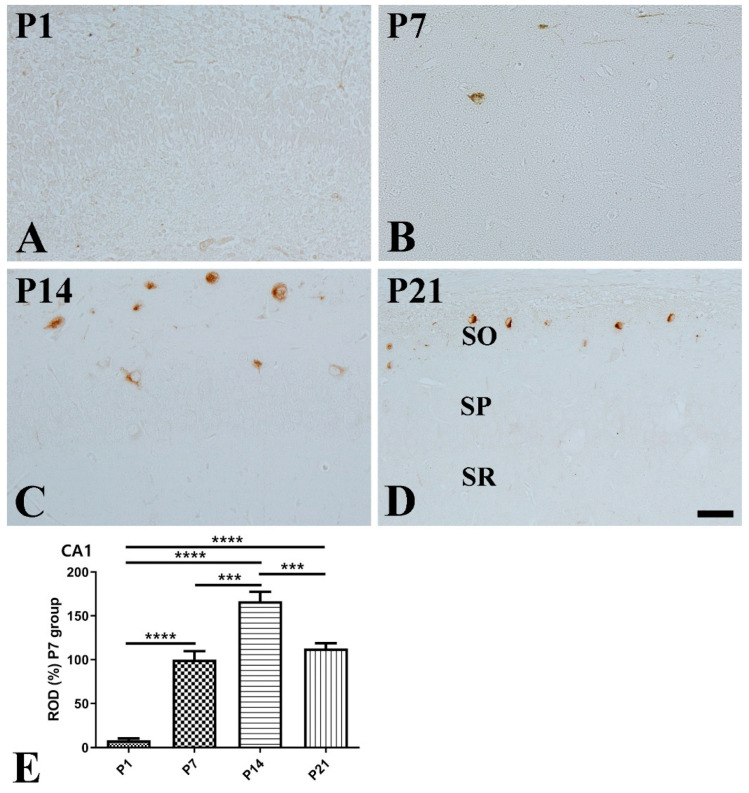
Immunohistochemical staining for somatostatin (SST) in the mouse hippocampal CA1 region at P1 (**A**), P7 (**B**), P14 (**C**), and P21 (**D**). SO stratum oriens; SP stratum pyramidale; SR stratum radiatum. Scale bar = 50 μm. (**E**): The number of SST-immunoreactive cells in the hippocampal CA1 region at P1, P7, P14, and P21 are expressed as a percentage of the values in the P7 group (*n* = 5 per group; *** *p* < 0.001 and **** *p* < 0.0001). All data are represented as the mean ± standard error of the mean.

CA1 region: At P1, SST-immunoreactive cells were rarely detected in the CA1 region (Figure 2A). At P7, SST immunoreactivity was markedly increased 11.96-fold compared with that at P1, and the majority of the SST-positive cells were detected in the stratum oriens (Figure 2B,E). At P14, SST immunoreactivity was significantly higher compared to that at P7, and a few SST-positive cells were detected in the stratum pyramidale (Figure 2C,E). At P21, SST immunoreactivity was significantly decreased compared to that at P14, and SST-positive cells were detected only in the stratum oriens (Figure 2D,E).

**Figure 3:**
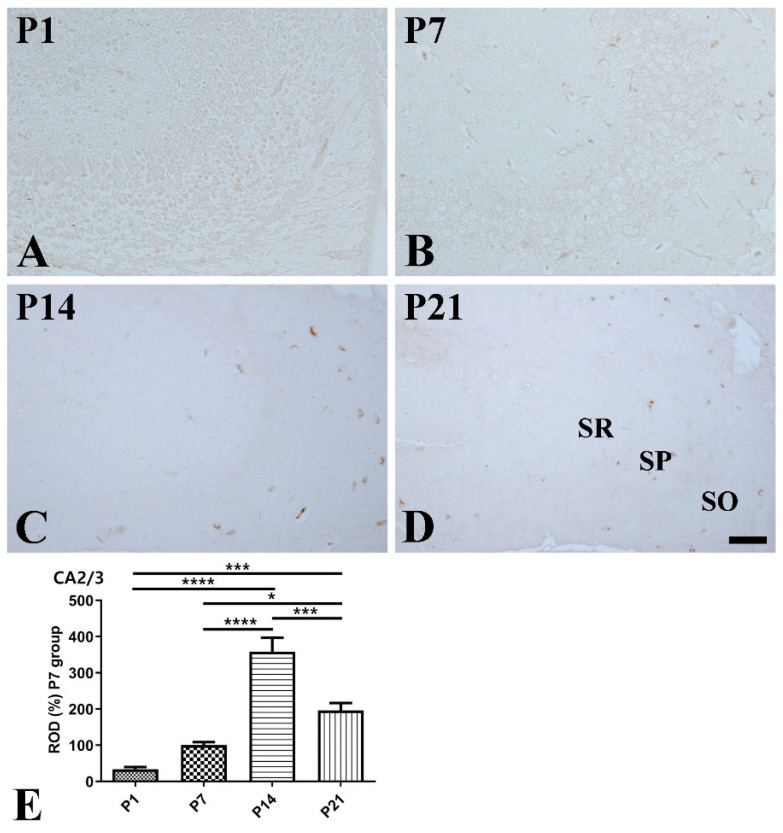
Immunohistochemical staining for somatostatin (SST) in the mouse hippocampal CA2/3 region at P1 (**A**), P7 (**B**), P14 (**C**), and P21 (**D**). SO stratum oriens; SP stratum pyramidale; SR stratum radiatum. Scale bar = 50 μm. (**E**): The number of SST-immunoreactive cells in the hippocampal CA2/3 region at P1, P7, P14, and P21 are expressed as a percentage of the values in the P7 group (*n* = 5 per group; * *p* < 0.05, *** *p* < 0.001, and **** *p* < 0.0001). All data are represented as the mean ± standard error of the mean.

CA2/3 region: At P1, SST expression was hardly detected in the CA2/3 region (Figure 3A). At P7, SST-immunoreactive cells were detected in the stratum oriens as well as the stratum radiatum (Figure 3B). At P14, SST immunoreactivity was significantly higher compared to that at P1 and P7, and SST-positive cells were mainly located in the stratum oriens (Figure 3C,E). At P21, SST-positive cells were mainly detected in the stratum oriens, but SST immunoreactivity was significantly decreased compared to that at P14 (Figure 3D,E).

**Figure 4:**
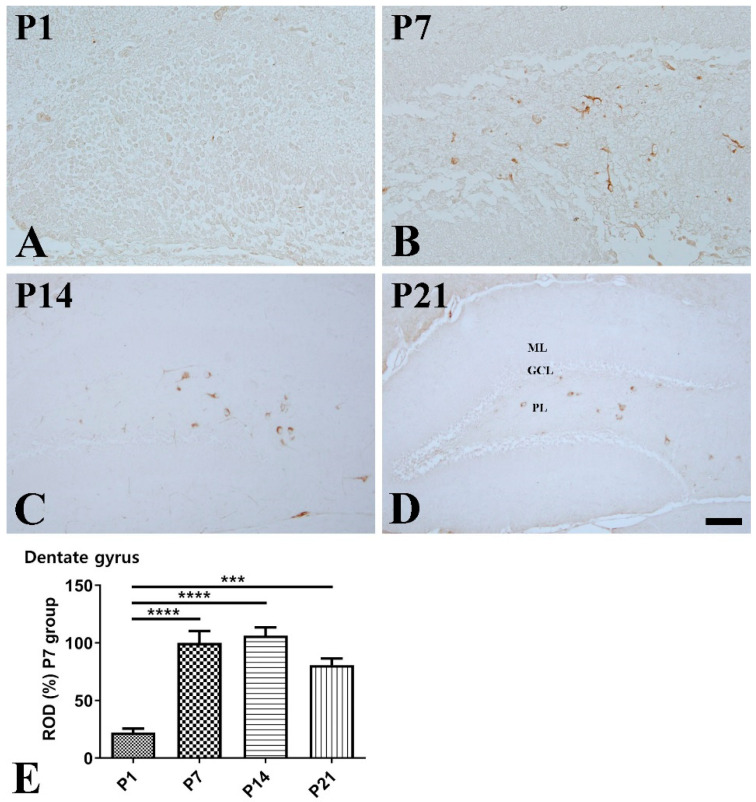
Immunohistochemical staining for somatostatin (SST) in the mouse dentate gyrus at P1 (**A**), P7 (**B**), P14 (**C**), and P21 (**D**). GCL granule cell layer; ML molecular layer; PL polymorphic layer. Scale bar = 50 μm. (**E**): The number of SST-immunoreactive cells in the dentate gyrus at P1, P7, P14, and P21 are expressed as a percentage of the value in the P7 group (*n* = 5 per group; *** *p* < 0.001 and **** *p* < 0.0001). All data are represented as the mean ± standard error of the mean.

Dentate gyrus: In the hippocampal dentate gyrus, the expressional pattern of SST was similar to that in the CA1-3 regions. At P1, SST-immunoreactive cells were rarely observed in the dentate gyrus (Figure 4A). At P7, SST-immunoreactivity was well-detected in the cell bodies and fibers in the polymorphic layer and significantly increased compared with that at P1 (Figure 4B,E). At P14, SST-positive cells were clearly observed in the polymorphic layer (Figure 4C). At P21, SST immunoreactivity was slightly decreased compared with that at P14 (Figure 4D,E).

### 3.3. Double Immunofluorescence of NeuN or GFAP with SST at P14

**Figure 5:**
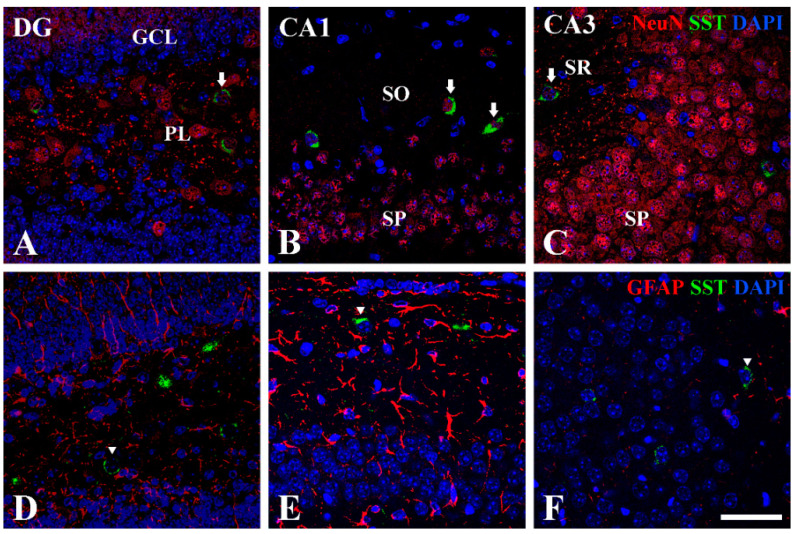
Double immunofluorescence staining for NeuN (red, (**A**–**C**)) and GFAP (red, (**D**–**F**)) with SST (green, (**A**–**F**)) in the hippocampal dentate gyrus, CA1, and CA3 regions at P14. DAPI is expressed as blue in all images. SST immunoreactivity is co-localized only with NeuN-positive cells (arrows), not GFAP-positive cells in the PL, SO, and SR. GCL granule cell layer; PL polymorphic layer; SO stratum oriens; SP stratum pyramidale; SR stratum radiatum. Scale bar = 50 μm.

In the hippocampal sub-regions, we performed double immunofluorescence staining for NeuN or GFAP with SST to find out which cells express SST. The results of double immunofluorescence staining showed that SST immunoreactivity was detected in NeuN-positive neurons, not in GFAP-positive astrocytes (arrows in Figure 5).

### 3.4. Effects of SST on Cell Proliferation and Neuroblast Differentiation in the Hippocampal Dentate Gyrus

**Figure 6:**
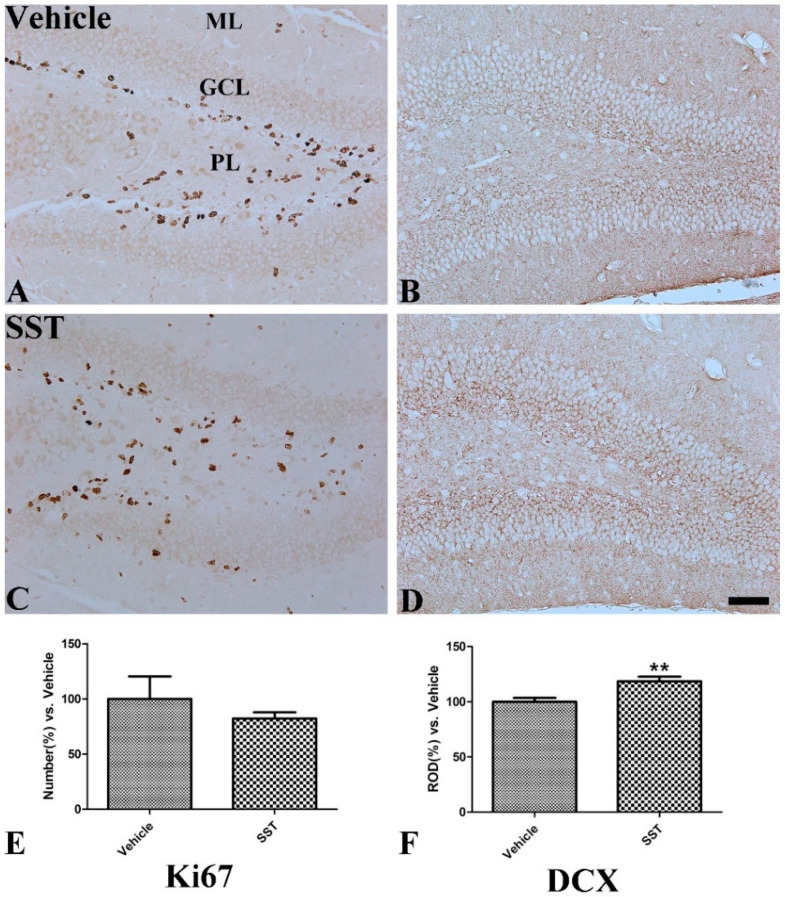
Immunohistochemical staining for Ki67 and DCX in the mouse dentate gyrus of the vehicle—(**A**,**B**) and somatostatin—(SST, (**C**,**D**)) treated groups. Scale bar = 50 μm. (**E**): The relative number of Ki67-positive neurons per section in the dentate gyrus of the vehicle- and SST-treated groups (**F**): The relative optical density of DCX-positive immunoreactivity in the dentate gyrus of the vehicle- and SST-treated groups, GCL granule cell layer; ML molecular layer; PL polymorphic layer, *n* = 5 per group; ** *p* < 0.01, significantly different from the vehicle-treated group. All data are represented as the mean ± standard error of the mean.

In the vehicle-treated group, Ki67-positive cells were mainly detected in the subgranular zone and polymorphic layer of the dentate gyrus (Figure 6A). In the SST-treated group, the mean number of Ki67-immunoreactive cells was decreased but, was not significantly changed compared to that in the vehicle-treated group (82.31% of the vehicle-treated group, Figure 6C,E). DCX immunoreactivity for differentiating neuroblasts was also observed in the subgranular zone in the vehicle-treated group (Figure 6B); however, in the SST-treated group, relative optical density for DCX was significantly increased (118.64% of the vehicle-treated group, Figure 6D,F).

## 4. Discussion

In the adult hippocampus, SST-immunoreactive cells were mainly detected in the stratum oriens and the polymorphic layer of the dentate gyrus [26,27], but SST and its receptors exhibit transient expression changes during brain development [28]. In the neocortex and the hippocampus, SST mRNA expression was detected at very low levels during a gestation period, but a progressive increase in SST mRNA expression was observed in postnatal animals [29]. In the present study, we observed the chronological and cell-based changes in SST expression in the mouse hippocampal sub-regions during the early postnatal period.

At P1, SST-positive cells were rarely detected in all the sub-regions of the hippocampus. The number of SST-positive cells markedly increased with time by P14 and decreased thereafter. At P7 and P14, SST-immunoreactive cells were well detected in the stratum oriens of the CA1-3 regions, as well as the polymorphic layer of the dentate gyrus. At P21, however, SST immunoreactivity was decreased in all the sub-regions of the hippocampus.

In our previous study, we observed the expression of DCX in the dentate gyrus during the early postnatal period, and the lamination of the dentate gyrus was defined from P7 by cresyl violet and NeuN staining; the dentate gyrus was assumed to be fully mature at P14 [9,25]. It was also reported that the rat hippocampus is completely mature at P15 [30,31], and the timing of the brain growth spurt, defined as the total brain weight gain as a percentage of the adult weight, was found to peak around birth in humans and at P7 in rats [32]; the volumetric development of the mouse brain is almost complete at P20, whereas the human brain volume reaches a similar development around 2~3 years of age [23]. In addition, we also observed the glucose transporter 3 (GLUT3), a main neuronal GLUT, immunoreactivity mainly observed in the DCX-immunoreactive neuroblasts with spatial expression from polymorphic and granule cell layers of the dentate gyrus to cells in the subgranular zone by P28 [25].

In the present study, we found that SST immunoreactivity in the hippocampal sub-regions transiently increased during P7 and P14, which nearly coincides with the period of postnatal cellular maturation in the mouse hippocampus [33]. An increase in SST immunoreactivity in reeler mice during the postnatal period suggests that SST may have a developmental role in the CNS [34]. Except for the hippocampus, SST shows transient high expression in the cerebellum and the retina during the prenatal and postnatal periods [12,35], and Forloni et al. [13] suggest that SST may have a morphogenic function. In the human brain, SST immunoreactivity has been found at a higher density during prenatal and perinatal periods and decreased from postnatal months 5 onwards [36].

In the present study, we administered SST to investigate its effects on neurogenesis in the dentate gyrus of P7 mice, as P7 is the dynamic period for investigating perinatal events [22,23,33], and postnatal development of the blood–brain barrier is not completed until P12 [24]. In addition, in the developing mouse brain, neural stem cells remain rich enough until P14, and they begin to differentiate into neural progenitor cells at this time [37]. It has been reported that the action of SST on its receptors negatively modulates cell proliferation [38], and the anti-proliferative effects of SST are mediated by the specific subtypes of SST receptors [39]. Many researchers have targeted the SST receptor-mediated pathway as an anti-cancer therapy due to its anti-proliferative and apoptotic effects on specific types of cancers [40,41,42]. In this study, we observed that the administration of SST in P7 mice for 7 days did not significantly decrease the number of Ki67-immunoreactive cells in the dentate gyrus. Ki67 is a marker for proliferating cells including newly generated neuroblasts, astrocytes, and microglia in the brain. However, in the present study, we could not elucidate what types of cells are decreased after SST teatment. The typing of cells needs to be elucidated to understand the roles of SST on cell proliferation in brain.

In this study, we observed that treatment of SST significantly increased neuroblast differentiation in the subgranular zone of the dentate gyrus. Leroux et al. suggested that transient expression of SST receptors during brain development is associated with morphogenetic activities [43], and SST expression decreases markedly with aging in the rodent hippocampus [44]. In the previous study, we observed a significant reduction in DCX-immunoreactive neuroblast in the mice dentate gyrus between P7 and P21. In addition, we observed the transition of neuroblasts into mature neurons in the granule cell layer of the dentate gyrus between P14 and P21 in mice [9]. Several studies have reported that interactions between SST- and G-protein-coupled SST receptors affect neurotransmission and proliferation but correlate with neuroblast differentiation [45]. In addition, high levels of SST in plasma or tumors are detected in neuroblastoma [46,47]. Recently, it was reported that SST is co-localized in cannabinoid CB1-receptor-immunoreactive cells in the hippocampus [48], suggesting a close relationship between SST and the CB1 receptor. The functional crosstalk between the cannabinoid CB1 receptor and the SST receptor 5 is closely associated with signal transduction of extracellular signal-regulated kinase and phosphoinositide 3-kinase pathways, which are known to enhance neuroblast differentiation [49,50].

SST is expressed in CNS and various peripheral organs, and about 30% of circulating SST is expressed in the brain [51]. It has been reported that circulating SST regulates expression of the hypothalamus-pituitary-adrenal axis, including adrenocorticotropic hormone [52]. Signaling mediated by glucocorticoid receptors in the hippocampus modulate expression of neurotrophic factors such as brain-derived neurotrophic factor (BDNF) and fibroblast growth factor 2, closely involved in neuroblasts differentiation [52]. In addition, BDNF signaling regulates SST expression [53], and SST also has an important role in bone morphogenetic protein signaling, which regulates tissue structure and differentiation of stem cells in the CNS and periphery [54,55].

In the present study, we confirmed that a transient increase in SST occurs in hippocampal sub-regions, and administration of SST modulates hippocampal neurogenesis during postnatal brain development. However, more studies about downstream signaling need to elucidate the exact roles of SST on hippocampal development.

## 5. Conclusions

SST-immunoreactive cells were abundantly detected in the hippocampal sub-regions of mice at P7 and P14 and decreased immunoreactivity was observed at P21. In addition, the administration of SST significantly increased DCX expression in the dentate gyrus. These results suggest that the transient increase in SST expression may be correlated with neuroblast differentiation in the hippocampus during postnatal development.

## Data Availability

Not applicable.
